# New Technique for Tramadol Dose Accuracy: Are Dosing Pumps the Solution?

**DOI:** 10.3390/ph19050796

**Published:** 2026-05-19

**Authors:** César Alas-Pineda, Carlos Coto-Tejeda, Dennis J. Pavón-Varela, Kristhel Gaitán-Zambrano, Jhacely Medina-Mejía, Gustavo Ferrer

**Affiliations:** 1 Department of Research and Development, Moxie Health Group, Hallandale Beach, FL 33009, USA; carloseduardocoto@gmail.com (C.C.-T.); dennisjpavon@gmail.com (D.J.P.-V.); kmelissag13@gmail.com (K.G.-Z.); jhacely2000@outlook.es (J.M.-M.); 2 Department of Pulmonary and Critical Care Medicine, Aventura Hospital and Medical Center, Aventura, FL 33180, USA; gferrer@pulmonary-institute.com

**Keywords:** dose, pump, tramadol, drops, chronic pain

## Abstract

**Background/Objectives**: Chronic pain represents a core global health burden and remains a leading cause of disability and reduced quality of life. Tramadol is a widely prescribed analgesic with a dual-opioid and non-opioid mechanism of action; however, safety concerns persist. This scoping review summarizes current evidence on tramadol pharmacology, dosing, and safety. **Methods**: A systematic literature search was conducted across main scientific databases to identify preclinical and clinical studies evaluating tramadol pharmacokinetics, safety, and dosing. **Results**: The same tramadol presentations have been used for several years, with a high overdose risk due to incorrect dosing measurements. Dosing pump devices represent a viable solution to improve dosing accuracy and minimize this risk. **Conclusions**: Tramadol drops seem like an easier administration pathway but require careful handling when taking the prescribed dose. A dosing pump in the medication bottle will improve dosing accuracy.

## 1. Introduction

Pain remains one of the most prevalent and challenging clinical conditions worldwide and represents an economic burden on healthcare systems. Chronic pain is a common condition in primary care and is defined as any continuous or recurrent pain that lasts more than 12 weeks in a clinical setting [1]. Pain is a biopsychosocial phenomenon; its management focuses on addressing its effects, maximizing function, and improving quality of life.

In recent years, the prevalence of chronic pain has shown an upward trend. Worldwide estimates indicate that its prevalence remains within a relatively narrow range, from 27.5% to 30.3% [2]. In the United States alone, 50 million individuals reported chronic pain in 2016 [3]. The National Center for Health Statistics reported that 20.4% of adults in the United States experienced chronic pain in 2019, increasing to 24.3% in 2023 [4].

As prevalence increases, so do the burdens that chronic pain involves. Work absenteeism becomes more frequent, and primary healthcare systems get overloaded. New treatments are being developed; their clinical integration necessitates careful evaluation due to the historical risk of substance abuse associated with opioid analgesics. New dosing strategies of preexisting drugs are being considered. This review aims to summarize the pharmacokinetics, clinical evidence, and future strategies for oral dosing pumps.

## 2. Methods

**Figure 1:**
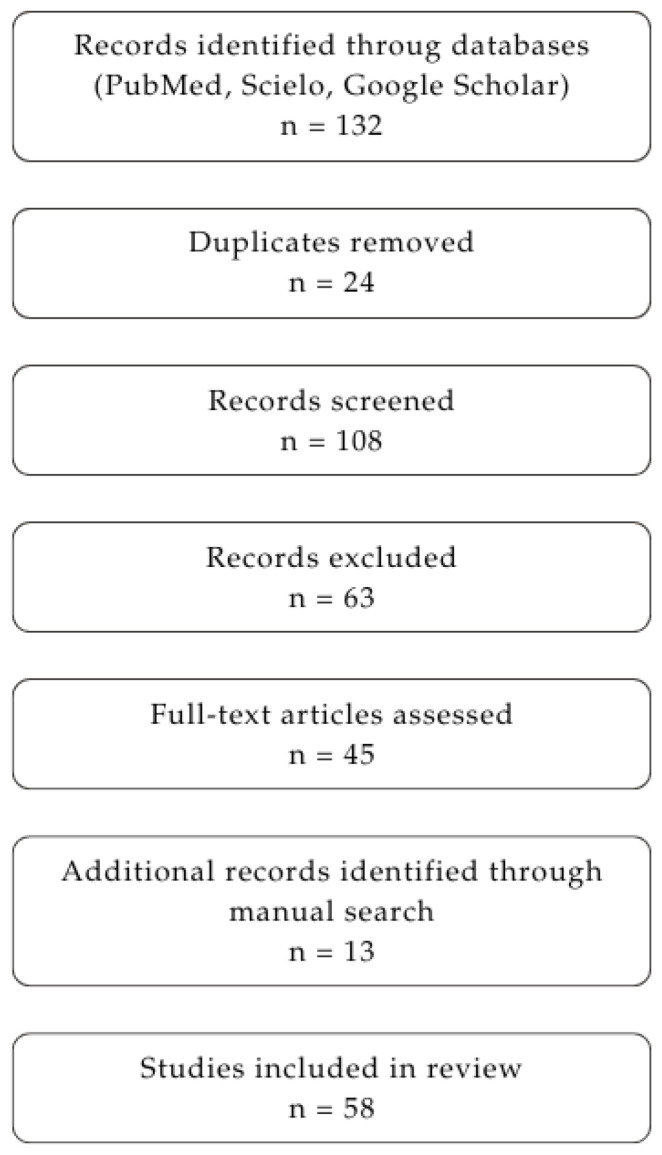
PRISMA flow diagram illustrating the identification, screening, eligibility, and inclusion of studies in the review.

We conducted a scoping literature review of the available evidence on tramadol pharmacology, dosing accuracy, adverse effects, and the potential role of pre-calibrated dosing devices in reducing administration errors, especially in the elderly population. The search was performed across PubMed, SciELO, and Google Scholar, prioritizing information from the last 10 years (2015–2026) to highlight the most recent available evidence. No language restrictions were applied. Key search terms included: tramadol, chronic pain, pharmacokinetics, drop formulation, pump, safety, and dose accuracy. Studies were qualified for inclusion if they reported data on the following: tramadol pharmacokinetics, mechanism of action, or pharmacodynamics; adverse effects, dosing regimens, and safety; drug delivery methods; and novel devices or technologies. A total of 132 records were identified (as shown in Figure 1). After the removal of duplicates (*n* = 24), 108 records were screened based on title and abstract. Of these, 63 records were excluded for failing to meet the inclusion criteria. A total of 45 full-text articles were assessed for eligibility. Additionally, 13 records were identified through manual searches and reference screening, bringing the total to 58 studies included in the final review. Data was extracted and synthesized, focusing on pharmacology, doses, safety, adverse effects, and new delivery technologies.

## 3. Results

Pain has consistently accompanied the human experience throughout history. The understanding of pain has evolved alongside the development of civilizations in every aspect. The understanding of pain evolved from a mystical-religious interpretation to a physiological imbalance [5]. Across several studies, multiple advances were made, including new terminology, the identification of pathophysiological pathways, and recognition of it as a disease in its own right. [6,7]. Although many efforts were made to treat pain, a safe and effective treatment is still needed, as demonstrated in the mid-1990s, when a marked increase in opioid prescriptions paralleled a rise in adverse public health outcomes [8].

In 2020, the International Association for the Study of Pain (IASP) modified its definition of pain. The new definition is “An unpleasant sensory and emotional experience associated with, or resembling that associated with, actual or potential tissue damage.” [9]. It is important to understand the pathophysiology of pain and how to assess it. The nervous system is the key point in this context, including the different types of neurons: sensory, interneurons, and motor. Nociceptors, a type of sensory neuron, detect chemical and physical stimuli. Each nociceptor has different types of axons (bundles of nerve fibers) that also detect stimuli. Aδ fibers mediate nociceptive input related to thermal and mechanical stimuli, while C fibers respond to a wide range of noxious signals (thermal, mechanical, and chemical) [10].

Pain transmission is the process by which nociceptors receive peripheral information and transmit it to the spinal cord. The first step is made by nociceptors when a stimulus is perceived, which generates the release of excitatory neurotransmitters in the dorsal horn. Then, second-order neurons are activated, and these neurons transmit information to the brain stem and thalamus via ascending pathways (the spinothalamic tract to supraspinal structures). In the thalamus, third-order neurons are located, and their function is to send information to the primary sensory cortex for pain perception [11].

Three main types of pain have been described. Neuropathic pain is any sensation caused by a lesion or disease of the somatosensory system. It may originate at different levels of the transmission pathway, presenting as burning, pressure, or electric shock sensations [12]. Nociceptive pain results from injury to non-neural tissue and is localized, yet it may be referred [13]. Nociplastic pain is characterized by altered nociception in the absence of clear evidence of tissue damage or somatosensory lesions. It is associated with increased sensitivity and symptoms such as allodynia, hyperalgesia, and spontaneous pain [14].

The first descriptions of opioids were made by the Sumerians, who portrayed opium poppy (Papaver somniferum, Papaveraceae) on a clay tablet [15]. In 1962 in Germany, the company Grünenthal GmbH produced Tramadol as an analgesic drug, and commercialized it in 1977 under the same name, “Tramadol” [16].

Addiction to opioids, or opioid use disorder as it is properly called, begins with the misuse of the drug. When used for an extended period or at high doses, it can lead to tolerance and dependence. Opioids release dopamine through the mesolimbic system, activating opioid receptors that inhibit the release of γ-aminobutyric acid (GABA), which regulates dopamine release. As a result, it creates euphoria and anxiolysis [15]. Among patients prescribed opioids, 0.6% develop an opioid use disorder [17].

### 3.1. Mechanism of Action

**Figure 2:**
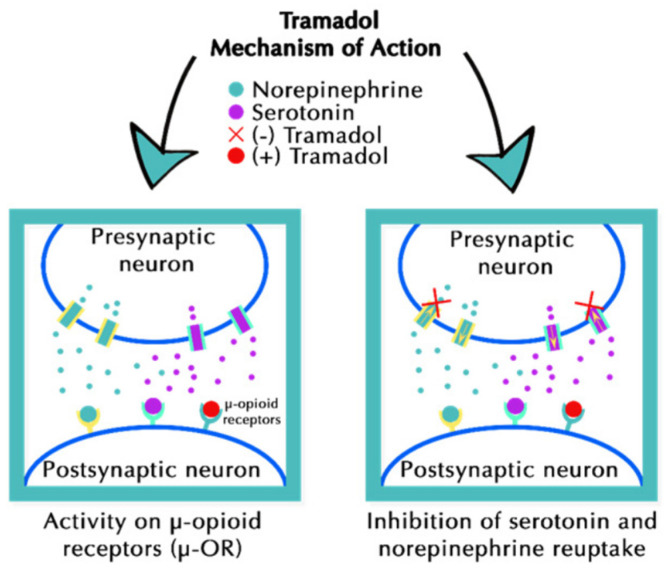
Dual mechanism of tramadol’s pharmacological activity in modulating pain perception. Reproduced from Reference [18] under CC BY 4.0 license.

Tramadol hydrochloride, chemically named as 2-[(dimethylamino)methyl]-1-(3-methoxyphenyl) cyclohexanol hydrochloride, with the molecular structure C_16_H_25_NO_2_, is a synthetic opioid of the phenylpropanolamine class [18]. It is composed of two pharmacologically active enantiomers. This produces analgesic activity through a dual mechanism that involves both opioid and non-opioid pathways. Its pharmacological action includes low-affinity binding to μ- and κ-opioid receptors, resulting in weak agonist activity; see Figure 2.

The (+)-enantiomer, in combination with its primary active metabolite (+)-O-desmethyltramadol (M1), displays central analgesic effects predominantly through agonism at the μ-opioid receptor. It produces analgesic effects without respiratory depression [19]. It simultaneously modulates monoaminergic neurotransmission by preventing the reuptake of serotonin and norepinephrine. Specifically, the (+)-tramadol produces an inhibition of the serotonin reuptake, and the (–)-tramadol inhibits norepinephrine [20]. This combined mechanism attenuates spinal nociceptive signaling [16]. This dual-action mechanism has been studied primarily in oncology and chronic pain. Although tramadol is a safer option, it has a less potent analgesic effect than a full agonist opioid, but it has the same risks, such as addiction and sedation [17].

Also, tramadol changes the perspective on how pain is perceived. This is possible through tramadol’s interactions with the regulation of glial cell activity, cytokine release, and prostaglandin E2 synthesis, as well as with α2-adrenergic receptors, neurokinin-1 receptors, and voltage-gated sodium channels. All of these are non-opioid pathways of pain perception [20].

Rubinstein et al. summarize the six pharmacologic properties of tramadol, including agonistic activity at μ-opioid receptors, weak inhibition of serotonin and norepinephrine reuptake, functions as an antagonism of serotonin 5-HT2C receptors, produces a competitive inhibition of muscarinic M1 and M3 receptors, a non-competitive antagonism of N-methyl-D-aspartate (NMDA) receptors, and a complex agonistic interaction with the transient receptor potential vanilloid 1 (TRPV1) [21].

### 3.2. Pharmacokinetics

Several pharmaceutical formulations of tramadol are available on the market, including capsules, tablets, syrup, cream, ointment, gel, drops, and parenteral formulations. Peak plasma concentration is reached within 2 h for capsules, 1.2 h for drops [22], and 5 h for sustained-release tablets; 20% is bound to plasma proteins [23].

Tramadol has a 75% oral bioavailability. It reaches peak plasma concentration in 2–3 h and has a half-life of 6 h. A stable plasma concentration is reached within 2 days when administered every 6 h [24].

Tramadol undergoes extensive hepatic biotransformation, such as demethylation, oxidation, and conjugation [25]. Over 70% is metabolized via cytochromes P450 (CYP), predominantly CYP2D6, and CYP3A4. During phase 1, CYP3A4 mediates N-demethylation to yield N-desmethytramadol, whereas CYP2D6 facilitates O-demethylation to form O-desmethyltramadol, which is the principal active metabolite responsible for the 40% of analgesic action [25,26].

Renal excretion accounts for 90% of tramadol. In addition, around 10–30% of tramadol is excreted as an unmetabolized drug, and nearly 60% is excreted as metabolites [23]. The metabolic pathway described depends on CYP2D6, which exhibits pharmacogenetic variability, a principal challenge in achieving a consistent therapeutic effect, as explained in the following section.

### 3.3. Variability and Challenges in Dosing

In addition to its pharmacokinetic characteristics, the clinical safety profile of tramadol depends on the accuracy of its dosing and administration. Medication administration errors are commonly associated with liquid preparations, especially among vulnerable populations. Liquid formulations account for 80% of pediatric home medication errors; the main reasons include confusion about measuring liquids, leading to over- or underdosing. To avoid these errors, dosing tools should be used instead of teaspoons, as they vary in size and shape [27]. In a cross-sectional study, Yin et al. recollected data from parents of children evaluated in the emergency room; 81.4% gave doses below the prescribed amount, whereas 18.6% gave doses above the prescribed amount. Additionally, parents who received a dosing tool were less likely to use a kitchen spoon. Caregivers who use a teaspoon or a tablespoon had twice the risk of making a dosing error [28,29]. Although dosing cups are effective and widely used, there is a higher risk of dosing errors when the cup is not held to eye level, as it may appear to be filled to a particular mark when it is not. A randomized controlled study reported fewer errors when the prescription specified whole numbers (5 mL vs. 2.5 mL) [30].

Moulis et al. evaluated 74 cases of adverse drug reactions secondary to tramadol. Thirteen were medication errors, and eight were with oral formulation. Two cases were due to drop-to-milligram variability as the doses in the prescription were in mg or in mg per day. One case of a 3-year-old boy ended in death due to critical adverse drug reactions, due to an error in drop counting the dose, and no evidence of polymorphism was reported [31].

CYP2D6 has multiple polymorphisms that influence tramadol bioavailability across populations, even when the same dose is prescribed. More than 150 genetic variations have been discovered. The hepatic metabolic rate in each individual is directly proportional to the analgesic and side-effect profiles of tramadol [24,26]. These genetic variations are classified as: poor metabolizers (PMs), intermediate metabolizers (IMs), normal metabolizers (NMs), and ultra-rapid metabolizers [32].

Polymorphisms vary across populations. PMs represent approximately 6–10% of the Caucasian population. This means that the analgesic effect of tramadol is reduced; they have approximately 20% higher plasma tramadol concentrations than NMs. In contrast, 28% of North Africans, Ethiopians, and Arabs and around 10% of Caucasians are considered ultra-rapid metabolizers. In this context, they produce higher plasma concentrations of O-desmethyltramadol (M1), the active metabolite, which increases opioid effects and toxicity [26]. O-desmethyltramadol has an approximately 300-fold higher affinity for μ-receptors than tramadol [33].

To determine an individual’s metabolizer status, two processes can be performed. It can be done by genotyping or phenotyping; both are crucial for improving response to sensitive CYP2D6 substrate drugs. Phenotyping is done by measuring the concentrations of the parent drug and the metabolite of interest (in this case, the active metabolite) in blood and/or urine. Then, concentration ratios are compared (parent/metabolite or vice versa) to predefined cut-off values that discriminate between genotype/phenotype groups [33].

This genetic variability intervenes in both overdosing and underdosing when doses of tramadol are administered [18].

In the CYTRAM study, De La Gastine et al. describe that poor metabolizers showed the lowest mean plasma concentration of O-desmethyltramadol. Also, the O-desmethylated tramadol/tramadol ratio at 24 and 48 h was lower than in non-poor metabolizers [33].

Ishitsubo et al. state that plasma concentrations of tramadol reached steady state approximately 24 h after administration, with no excessive accumulation observed. The peak maximum plasma concentration was 300 ng/mL, compared with the minimum measurement of 120 ng/mL [34]. Table 1 summarizes limitations and key information derived from available data.

### 3.4. Side Effects

As with any other medication, patients may experience side effects ranging from minor to severe. Side effects such as nausea, vomiting, dry mouth, sweating, dizziness, and constipation may be present [41,43]. It is well known that tramadol may cause dependence, and withdrawal symptoms can emerge one week after tramadol was first administered for musculoskeletal pain [44].

Additionally, tramadol has been linked to a decreased seizure threshold, mainly in patients suffering brain trauma or who have seizure activity secondary to hypoxia, due to an inhibition of GABA [24,45]. Also, a prolonged QT interval is possible. Tramadol increases the risk of serotonin syndrome, especially when combined with a selective serotonin reuptake inhibitor (SSRI) [24,46].

Recently, attention has been drawn to a less common adverse effect: tramadol-induced immunosuppression. This effect is described in two main mechanisms. The first mechanism acts directly on μ receptors expressed on T lymphocytes, macrophages, and natural killer (NK) cells. This inhibits lymphocyte proliferation, the secretion of proinflammatory interleukins, and NK cell-mediated cytotoxicity. Conversely, the hypothalamic–pituitary–adrenal axis and modulation of the sympathetic nervous system are activated. This activation leads to an exacerbation of corticosteroid (glucocorticoid) and catecholamine release, producing an inhibition in the synthesis of proinflammatory interleukins while boosting the release of anti-inflammatory interleukins like Transforming Growth Factor Beta (TGF-β) and IL-10 [47].

De Canecaude et al. found an association between the administration of tramadol and hyponatremia. In this study, 225,575 individual case safety reports with tramadol, 815 reported hyponatremia, especially in patients older than 75 years old [48]. Other metabolic side effects include: hypoglycemia and increased anti-coagulant effect [41].

Addiction might be the most well-known side effect of tramadol and arises from its mechanism of action, in which the reward system is exacerbated, leading to a tendency to abuse. Additionally, it blocks the serotonin transporter (SERT), producing an ejaculation delay in men who already face premature ejaculation. Prolonged use can lead to poor sperm quality [49]. To prevent misuse, several regulations have been put in place due to a higher risk of abuse in its oral presentations. In 2017, the “past-year misuse” of total prescriptions was 4.6%, and 9.2% of those reported use, whereas in 2024, the “past-year misuse” was 8.0%. Tramadol is categorized as a Schedule IV drug, alongside alprazolam [50,51,52].

### 3.5. Dosing Regimens

In the general population, a standard therapeutic dose is given. Orally: 50 mg; rectally: 100 mg; parenterally: 50–100 mg. The maximum recommended daily dose is 400 mg. Given that the toxic blood concentration is 1 mg/L, the lethal concentration is 2 mg/L, and the therapeutic blood concentration is 0.1–0.3 mg/L. A small concentration of tramadol has been detected in breast milk, and it can pass the placental barrier [53].

Tramadol drops are another option to treat acute or chronic pain. Although it might seem like an easier administration pathway, dosage needs to be considered. It is vital to note that different oral presentations of tramadol drops do not have the same equivalence in drops per milliliter and, therefore, milligrams of tramadol per drop. Presentations can vary from 40 drops = 1 mL = 100 mg to 28 drops = 1 mL = 50 mg [38]. In Chile, Martínez et al. report that all presentations indicate that 1 mL of solution contains 100 mg of tramadol. Still, there is no standardization for the equivalence between the number of drops and 1 mL. In these situations, specifying the brand, milligrams per dose, and number of drops is key to preventing underdosing or, in the worst case, overdosing [39], as shown in Table 2. A pediatric presentation is available, in which 10 drops = 25 mg; this can also be used in elderly patients, especially in those with cognitive deficits [41].

In older patients, an oral starting dose using immediate release or subcutaneous injection is as follows: 12.5–25 mg every 4–8 h. When a prolonged-release form is used, the recommended dose is 50–100 mg every 12 h. For patients >75 years old, elimination may be prolonged; therefore, the dosage interval should be extended, if necessary, to a maximum of 300 mg/day. Increasing doses can result in dependence; a dose of 800 mg/day can generate the same dependence as morphine [45]. Comorbidities such as hepatic impairment (HI) and renal impairment (RI) need a reduced dose and a wider interval between each dose. In this case, the maximum dose is 200 mg/day [41].

### 3.6. Vulnerable Population

Bermúdez et al. reported a case of acute tramadol poisoning in a 4-month-old female infant in Ecuador, after receiving 24 drops of tramadol, 12 drops in each dose, instead of the prescribed paracetamol drops. The patient presented with classical signs of opioid toxidrome and was admitted for treatment and discharged with complete clinical recovery, highlighting the fact that tramadol is contraindicated in patients <12 years. This case represents how a pharmacy dispensing error can turn into a life-threatening overdose in the absence of a pre-calibrated dosing device [35].

Škvarč describes several recommendations for HI and RI patients. In RI, based on an Estimated Glomerular Filtration Rate (eGFR) of 30–89 mL/min, any opioid can be prescribed, considering each patient as an individual, monitoring clinical efficacy and side effects, and starting the dose lower than usual. Tramadol is not prescribed to those with an eGFR < 30 mL/min or to those undergoing hemodialysis. In contrast, HI patients, when opioids are administered, should be under strict monitoring, and doses can be increased cautiously due to the high risk of developing hepatic encephalopathy, opioid overdose, or constipation. Doses depend on the HI classification. For Child A, doses start at 50 mg and can reach a maximum of 200 mg/day. For Child B and Child C, doses start at 25 mg, and can reach a maximum of 100 mg/day [54].

In a European multidisciplinary consensus, prescribing tramadol to older patients requires particular consideration. Several factors contribute to the decision of prescribing an opioid in patients older than 75 years, such as confusion and delirium, neglect from caregivers, and proper age-related changes [41].

### 3.7. Safety Precautions

In some cases, an increased dose of tramadol might cause the death of the person. The authors believe the cause of death is due to the active metabolite, O-desmethyltramadol. Death can be caused when tramadol is administered with other medications that have a potential interaction, such as barbiturates, benzodiazepines, antidepressants, and alcohol. Tramadol is sometimes used to assist in suicide, as only one high dose is needed to produce respiratory depression. This is caused by overstimulation of the mu receptors, leading to decreased acetylcholine levels in the medulla and disrupting the response to CO_2_ [45]. Its administration is contraindicated in children under 12 years and pregnant women [24].

As a turning point, urologists used tramadol to treat premature ejaculation based on the same mechanism of action discussed previously, the inhibition of serotonin and noradrenaline reuptake in the central nervous system. Doses of 25 mg or 50 mg have been used with positive results. It is recommended to use an on-demand schedule rather than a daily one as it reduces the likelihood of side effects and dependence [55].

Ferron et al. describe how a patient with obesity receiving a CYP2D6 inhibitor can affect their metabolism, plasma concentration, efficacy, and toxicity of tramadol. He reported that tramadol concentrations were higher in patients receiving CYP2D6 inhibitors; however, this did not translate into an increased risk of toxicity, as O-desmethyltramadol (M1) concentrations were lower. Out of the five tramadol metabolites (M1, M2, M3, M4, M5), M5 had the highest concentration. Therefore, the analgesic effect is reduced, since it depends on a high concentration of M1. On the contrary, obesity and non-alcoholic fatty liver disease (NAFLD) are related to a decrease in CYP2D6 and CYP3A4 activity, which is why patients with obesity have high tramadol concentrations. NAFLD is associated with reduced HDL cholesterol and CYP3A4 activity, leading to decreased M3 concentrations. An inverse relationship between glycemia and tramadol metabolites was reported. With high glycemia, concentrations of M2 and M3 are decreased. This is consistent with the literature, which indicates that type 2 diabetes reduces the activity of CYP3A4 and CYP2B6 [56].

When using a drop presentation, it is necessary to know the equivalence between drops and mg. For a 5% tramadol solution, 20 drops are measured in a 1 mL dropper, 20 drops in 50 mg, so the recommendation is to start with 5 or 10 drops 4 times a day [40].

The authors recommend a starting dose of 200 mg/day. Depending on the response, it can be gradually increased to a maximum of 400 mg/day when treating diabetic neuropathy-induced pain. Due to dosing variations and the risk of over- or underdosing, precise drug administration is vital, particularly in drop formulations.

To ensure medication is administered with high safety standards and precise dosing, a new approach has been developed. To reduce the margin of error, a calibrated dosing pump connected directly to the tramadol drops bottle provides an effective solution. This fixed-volume pump mechanism delivers a pre-measured dose in every actuation, eliminating counting errors associated with the manual administration of drop medications. The ergonomic design and a low-force actuator mean it is easy to use by the vulnerable population. In contrast to manual drop counting, where dosing accuracy depends on user technique, fluid viscosity, measuring tool, and brand variation, a pre-calibrated dosing device is engineered to deliver a pre-measured dose in every actuation, theoretically reducing these sources of variability. However, quantitative data on dosing accuracy and pre-calibrated pumps remain lacking and represent a gap for future bench and human factors studies.

### 3.8. Scope and Limitations of Dosing Pump Devices

The safety profile of tramadol can be characterized into two distinct risk categories that should be considered when evaluating potential interventions. The first category includes dosing count errors, such as drop-to-milligram equivalence across commercial formulations, inexact drop counting, and caregiver drop-administration errors [31]. These factors are theoretically modifiable by implementing dosing technologies, such as pre-calibrated dosing devices, which may reduce the risk of drop counting errors.

The second category includes proper pharmacological and clinical risks that do not relate to accurate doses. Some factors that comprise this category are as follows. Pharmacogenetic variability in CYP2D6, which is in charge of phase 2 of the drug metabolism, determines the individual’s metabolizer status; poor metabolizers experience a reduced therapeutic effect, whereas ultra-rapid metabolizers produce higher plasma concentrations of the active metabolite, increasing the risk of opioid toxicity [26]. Additionally, altered pharmacokinetics in RI and HI patients require dose adjustments [41]. The drug–drug interactions with CYP2D6 and CYP3A4 inhibitors, or SSRIs, may lead to serotonin syndrome [46]. Furthermore, a reduction in the seizure threshold occurs in patients with brain trauma or hypoxic seizure history, and opioid dependence with prolonged use [45]. In addition, contraindications in specific populations, including children under 12 years of age, pregnant women, and breastfeeding mothers, may produce withdrawal effects in neonates [57].

Dosing pump devices address only the first category, omitting metabolizer status, adverse effects, pharmacokinetic alterations, drug–drug interactions, and contraindications. An effective safety profile requires a multilayered approach that integrates all these factors to reduce potential risks and side effects. Therefore, a pre-calibrated device may reduce drop-counting inaccuracy, but it does not address the intrinsic pharmacological and clinical risks.

## 4. Future Innovation for Drug Delivery

In the future, new devices need to be implemented for use when dosing oral medication, especially opioids, making it easier and safer for administration, more so, in children and elderly patients.

Tramadol drops represent an effective route of administration, particularly in patients undergoing oncology pain management and those who require enteral tube administration. This formulation offers a minor incidence of constipation [58]. It has been used in multiple conditions, such as the postoperative period after a thymectomy; in this scenario, 15 drops every 8 h were used [37]. Dose regimens may differ based on the clinical condition, specifically, in the treatment of cauda equina syndrome, a severe neuraxial anesthesia complication, a dose of 8 drops (equivalent to 20 mg) every 8 h was prescribed [36]. Tramadol drops require careful handling. In every dose, extra drops may result in a fatal overdose. The development of a specialized container configuration or a dedicated dosing device is essential, as errors with similar oral drop medications have been documented, as it did in a 4-month-old patient in Ecuador [35].

Several dosing errors may occur during the administration of tramadol drops, such as adding an excessive number of drops, failing to account for the drug’s concentration, or misinterpreting preparation instructions. These hazards are extremely important given that a 10 mL bottle of tramadol drops with a concentration of 100 mg/mL contains a total of 1000 mg of tramadol, a potentially lethal dose, especially in pediatric population, in which one drop contains 2.5 mg. Misunderstandings may occur, for example in the scenario of assuming that 15 drops are equivalent to 15 mL, in which case, 15 drops will deliver 37.5 mg [42]. To prevent these errors, a special pump or delivery mechanism should be installed in every tramadol bottle to reduce the risk of lethal overdose.

New-generation precise dose systems may integrate artificial intelligence or connect to a mobile application to enhance dosing accuracy and patient safety. These advances could facilitate real-time recording of each administered dose through logged actuation events, ensuring accurate dosing. Also, these systems may notify dosing errors such as overdose, subdose, or double doses, and alert in the case of an inadequate prompter activation. The incorporation of dose-locking mechanisms using polypropylene-based materials may help prevent dose administration errors.

## 5. Advantages and Limitations

This review has several limitations. The objective was to analyze available data, which limits the ability to estimate dosing errors. Actual evidence about tramadol drop-counting errors is limited. The available evidence consists of case reports, pharmacological reports, and reviews, but no clinical trials have been conducted.

No evidence of pre-calibrated dosing devices was identified. Based on this, the use of these devices is based on indirect evidence. Additionally, the variance in tramadol drops formulations, concentrations, and dosing regimens limited the ability to standardize accurate ml-to-mg doses.

## 6. Conclusions

This review highlights an important safety gap in the formulation of tramadol oral drops: the lack of standardized drop-to-milligram equivalence across commercially available presentations, which may increase the risk of dosing errors, especially in vulnerable populations such as pediatric patients and the elderly. Pre-calibrated dosing pumps represent a promising solution to address this gap, as fixed-volume mechanisms could theoretically reduce dosing errors compared to manual administration and counting. However, direct evidence related to tramadol is currently lacking, and the information presented in this review is based on indirect sources, including case reports, pharmacological analyses, and descriptive reviews of alternative liquid drug delivery systems. To date, no bench, usability, or clinical studies specifically assessing pre-calibrated dosing devices for tramadol have been identified.

Therefore, dosing pumps constitute a promising approach; claims about improved safety profile, reduced adverse effects, or higher dosing accuracy cannot yet be made. Future studies should focus on bench studies to determine the accuracy and precision of dosing pump prototypes using tramadol formulations, usability studies in vulnerable populations, and experimental clinical studies comparing dosing devices, dosing errors, and adverse effects related to standardized drop-to-milligram equivalence. Until this data is available, dosing pumps should not be considered a proven solution, but rather a candidate approach requiring rigorous evaluation.

## Figures and Tables

**Table 1:** Tramadol dosing errors.

Author (Year)	Country	Study Design	Population	Formulation	Dosing Outcome	Findings	Limitations
A. Case reports—Direct evidence on tramadol drops							
Bermúdez et al. (2021) [35]	Ecuador	Case report	Infant (4 months)	Tramadol drops	Overdose	Overdose due to drop-counting errors.	Isolated case
Merino-Urrutia et al. (2018) [36]	Chile	Case report	Adult	Tramadol drops	Dosing reports	Inconsistency in dosing regimens. 8 drops = 20 mg.	No dosing errors occurred
Oreamuno et al. (2019) [37]	Costa Rica	Case report	Adult	Tramadol drops	Dosing reports	No drop-to-milligram equivalence reported. Use of dosing regimens: 15 drops q8h.	No dosing error reported
B. Narrative and clinical guidance—Direct evidence on tramadol drops							
Catanzatiti (2024) [38]	Argentina	Brief communication	General	Tramadol drops	Variability in drop to mg equivalence	Increased risk of dosing errors (40 drops = 100 mg vs. 28 drops = 100 mg).	No clinical outcomes. Descriptive only
Martínez et al. (2017) [39]	Chile	Observational	Patients discharged from the ER	Tramadol drops	No drop-to-ml equivalence	Specifications on brand, mg, drops, and schedule need to be clarified to prevent dosing errors.	No clinical AE was described. Emphasizes the need for dosing standardization
Quintian (2018) [40]	Argentina	Clinical guidance	Musculoskeletal pain patients	Tramadol drops	Inconsistency in drop-to-milligram doses	Tramadol 5%. 20 drops = 50 mg. high risk of dosing errors.	No clinical outcome evaluation
C. Expert reviews—Indirect evidence							
Pickering et al. (2024) [41]	International review	Review	Elderly patients	Multiple formulations	Administration challenges	Elderly patients are at a higher risk of dosing errors due to cognitive impairment and altered pharmacokinetics.	No evaluation of drop counting errors
D. Experimental studies—Indirect evidence							
Kluger et al. (2016) [42]	Australia	Experimental study	Nursing surgical staff	Tramadol capsule dispersion	Accurate dose preparation	Dosing errors in oral formulations. Higher risk in formulations without standardization.	No specific drop formulation data

Abbreviations: ER, emergency room; mg, milligrams; AE, adverse effects.

**Table 2:** Drop-to-milligram variation.

Brand	Country	Number of Drops in 1 mL	Milligrams in 1 mL
Tramustin [38]	Argentina	40 drops	100 mg
Calmador [38]	Argentina	30 drops	5 mg
Lixidol [38]	Argentina	24 drops	100 mg
Ultracalmans [38]	Argentina	28 drops	50 mg
Manol [39]	Chile	40 drops	100 mg
Zaledor [39]	Chile	22 drops	100 mg
Tral [39]	Chile	25 drops	100 mg

Abbreviations: mg, milligrams.

## Data Availability

The original contributions presented in this study are included in the article. Further inquiries can be directed to the corresponding author(s).

## Abbreviations

The following abbreviations are used in this manuscript:

IASP	International Association for the Study of Pain
GABA	γ-aminobutyric acid
NMDA	N-methyl-D-aspartate
TRPV1	Transient Receptor Potential Vanilloid 1
CYP	Cytochromes
PMs	Poor Metabolizers
IMs	Intermediate Metabolizers
NMs	Normal Metabolizers
NK	Natural Killers
SERT	Serotonin Transporter
HI	Hepatic Impairment
RI	Renal Impairment
EGFR	Estimated Glomerular Filtration Rate
NAFLD	Non-Alcoholic Fatty Liver Disease
